# Silver nanoparticle-human hemoglobin interface: time evolution of the corona formation and interaction phenomenon

**DOI:** 10.1186/s40580-017-0122-1

**Published:** 2017-10-30

**Authors:** A. K. Bhunia, T. Kamilya, S. Saha

**Affiliations:** 1 Department of Physics & Technophysics, Vidyasagar University, Paschim Medinipur, 721102 India; 2 Department of Physics, Government General Degree College at Gopiballavpur-II, Beliaberah Jhargram, 721517 India; 3 Department of Physics, Narajole Raj College, Paschim Medinipur, 721211 India

**Keywords:** Human hemoglobin, Silver nanoparticles, Corona, DLS, HRTEM

## Abstract

In this paper, we have used spectroscopic and electron microscopic analysis to monitor the time evolution of the silver nanoparticles (Ag NP)–human hemoglobin (Hb) corona formation and to characterize the interaction of the Ag NPs with Hb. The time constants for surface plasmon resonance binding and reorganization are found to be 9.51 and 118.48 min, respectively. The drop of surface charge and the increase of the hydrodynamic diameter indicated the corona of Hb on the Ag NP surface. The auto correlation function is found to broaden with the increasing time of the corona formation. Surface zeta potential revealed that positively charged Hb interact electrostatically with negatively charged Ag NP surfaces. The change in α helix and β sheet depends on the corona formation time. The visualization of the Hb corona from HRTEM showed large number of Hb domains aggregate containing essentially Ag NPs and without Ag NPs. Emission study showed the tertiary deformation, energy transfer, nature of interaction and quenching under three different temperatures.

**Electronic supplementary material:**

The online version of this article (doi:10.1186/s40580-017-0122-1) contains supplementary material, which is available to authorized users.

## Introduction

Recently metal nanoparticle has shown its potential in diagnosis and therapy, particularly drug delivery, gene therapy, biosensor, bio imaging [1–5]. There are few successful applications of nanomaterials in biomedical domain include bacteria detection, alzheimer disease, early detection of cancer, protein fibrillation and so on. The silver nanoparticle (Ag NP) is used widely in environment, food and cosmetics industry [6–9]. Ag NP related products are used in technology, including biomedical and pharmacological applications [10, 11]. Small size silver nanoparticles (1–50 nm) exhibits larger surface area compared to its volume. This relatively large surface area increases their free energy and reactivity, which in many instances also increases toxicity [12]. The toxicity can limit the use of Ag NP in biology unless the nano-bio interaction is fully understood. Thus the fundamental question related its safety issue to health science, remains numerous challenges. Within this field, an area that has been largely unexplored is the interaction of metal nanoparticles with proteins [13–16]. Here, we used human hemoglobin (Hb) as model protein for study the interaction with Ag NPs. Hemoglobin is a tetrameric, globular and oxygen carrier protein. It composed of four subunit among which two are identical α-chains (α1, α2 contain 141 amino acid residues each) and two identical β-chains (β1, β2 each contain 146 amino acid residues) with each subunit has one oxygen binding heme-pocket [17–19]. Hb is rich of α-helix in native state [20]. It has various spectral signatures in electronic as well as vibrational spectroscopy [21]. When nanosize particles contact with Hb, Hb coronas are formed on NPs. Hb corona changes the surface properties of nanoparticles and governs the interaction between nanoparticles and Hb [14–16]. It causes Hb misfolding, which is intimately related to protein-mediated diseases [22]. In 1962, Leo Vroman showed that adsorption (which leads to corona) of blood proteins to a nanoparticle surface is time dependent [23].

In this paper the corona formation and structural deformation of human hemoglobin (Hb) induced by the assembly on silver nanoparticle (AgNP) surface were studied in time dependent manner by various spectroscopic techniques and electron microscopic method. The kinetic of the corona of Hb to the metalic Ag nanoparticle surface showed by the kinetic change in the LSPR of Ag nanoparticles and surface Zeta potential study along with dynamic light scattering (DLS) measurement. The circular dichroism (CD) spectra of the Hb–AgNPs bioconjugate system showed that AgNPs could dynamically induce the conversion of α-helix to β-sheet structures and deformation of the hemoglobin structure. The corona formation was studied by using DLS and zeta potential measurement.

## Experiment

### Synthesis of silver (Ag) nanoparticles

Here, we used a simple wet chemical method for Ag NPs synthesis [24]. Silver nitrate (AgNO_3_) and sodium borohydride (NaBH_4_) is used. Concisely, 40 mL, 20 mM AgNO_3_ solution was included drop wise into 60 mL, 100 mM of NaBH_4_ solution that had been chilled in an ice bath. Here, NaBH_4_ is a reducing agent which reduces the silver from AgNO_3_. The solution turned brighter yellow after all of the AgNO_3_ was added. The mixture was stirred by magnetic stirrer. The entire addition took ~ 30 min, after which the stirring was stopped. The clear yellow colloidal silver particle is stable at room temperature and stored in a transparent vial.

## Results and discussion

### Physicochemical characterization of silver nanoparticles

**Fig. 1:**
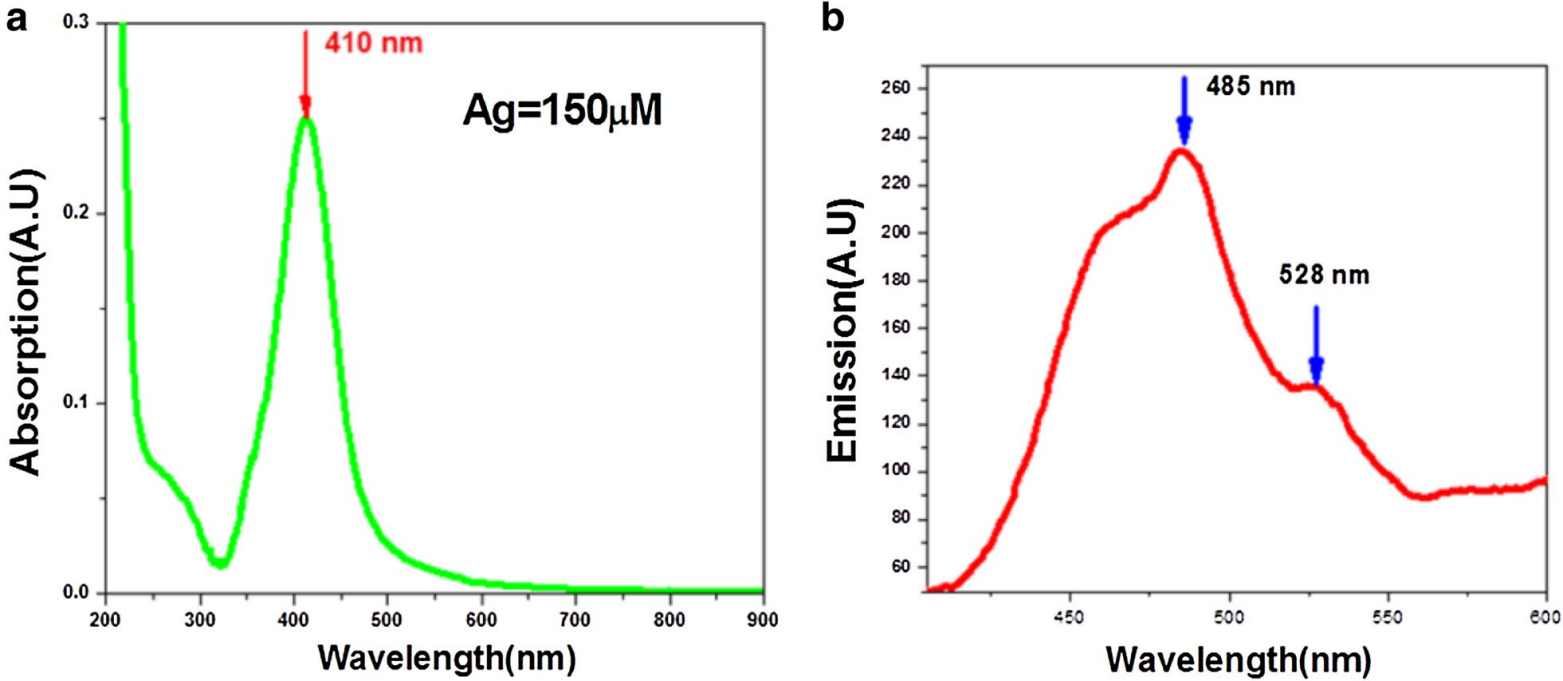
**a** Absorption spectra of pure Ag NPs. **b** Emission spectra of pure Ag NPs

**Fig. 2:**
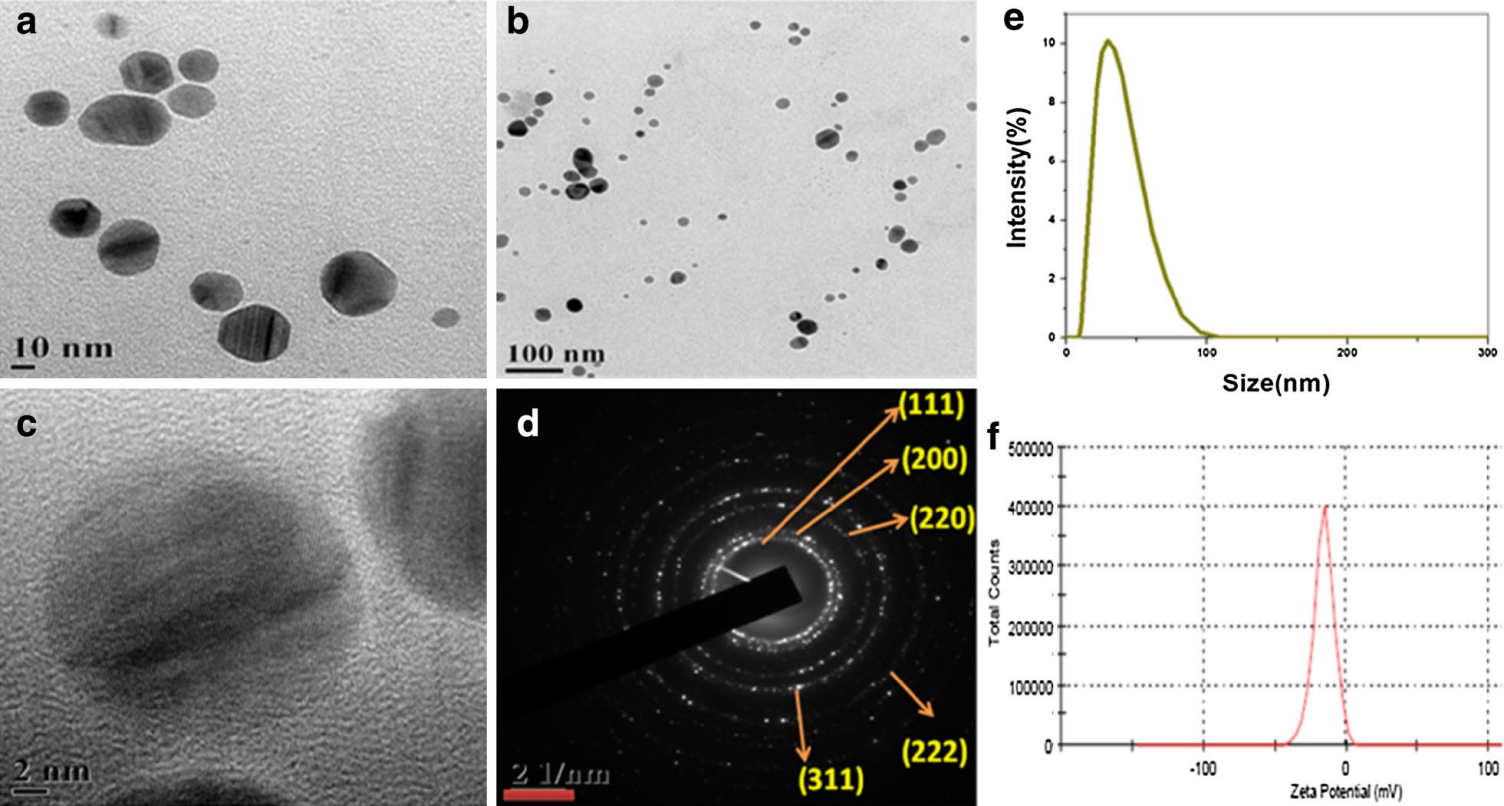
**a**, **c** HRTEM image of pure Ag NPs in 10 nm scale and 2 nm scale respectively. **b** TEM image of pure Ag NPs in 100 nm scale. **d** SAED pattern of Pure Ag NPs. **e** DLS of pure Ag NPs. **f** Zeta potential curve of pure Ag NPs

The absorption spectrum of Ag NPs in Fig. 1a shows strong absorption in visible range at 410 nm due to the surface plasmon resonance (SPR) [25, 26]. A typical emission of Ag NPs is observed at ~ 485 and 528 nm with an excitation wavelength 400 nm (Fig. 1b) [27]. The origin of the emission is due to the assistance of d band electrons of the Ag NPs on absorption of the incident photon energy, to higher electronic states in the sp-band. The less intense emission peak at 528 nm is due to the presence of the surface states of the nanoparticles [28]. As shown in Fig. 2a–c TEM images confirmed that the synthesized AgNPs were spherical. The size ranges from 8 to 25 nm with average size 15 nm and average number of silver atoms per nanoparticle is 10.4 × 10^4^. SAED pattern (Fig. 2d) shows crystalline nature of the nanoparticles with presence of the (111), (200), (220), (311), (222) crystal planes in the FCC phase of the unit cell. The average hydrodynamic diameter for Ag NP was 30 nm (Fig. 2e). Surface charge analysis revealed that AgNP had a net negative charge [29]. Ag NPs exhibited − 15.5 mV zeta potential (Fig. 2f).

### Effect of silver nanoparticles (Ag NPs) concentration on the hemoglobin (Hb) absorption

**Fig. 3:**
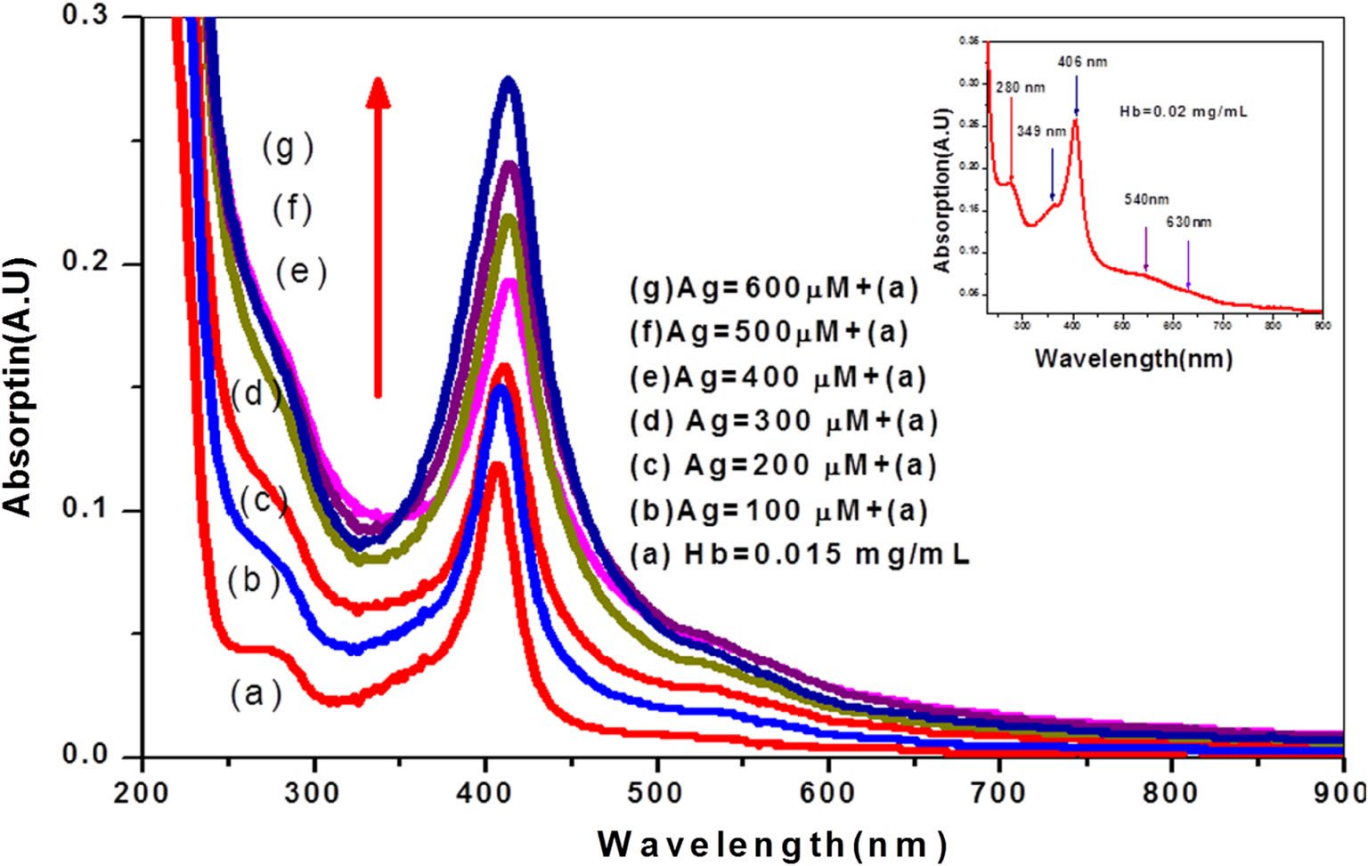
Absorption spectra of Hb-Ag NPs complex with (b) C_Hb_ = 0.015 mg/mL and C_ZnO_ = 100 µM. (c) C_Hb_ = 0.015 mg/mL and C_ZnO_ = 200 µM. (d) C_Hb_ = 0.015 mg/mL and C_ZnO_ = 300 µM. (e) C_Hb_ = 0.015 mg/mL and C_ZnO_ = 400 µM. (f) C_Hb_ = 0.015 mg/mL and C_ZnO_ = 500 µM. (g) C_Hb_ = 0.015 mg/mL and C_ZnO_ = 600 µM 600 µM, inset or (a) is the absorption of pure Hb (0.02 mg/mL)

The absorption spectrum of Hb (inset of Fig. 3) shows various electronic bands. The band at 280 nm arises due to π–π* transition of aromatic amino acid residues. The bands at 349, 406 nm are ε band and Soret band respectively. The other bands at 540 and 630 nm are oxy-band and Q-band respectively [24, 30–33]. The appearance of strong band at 406 nm (Soret band) reflects that the Hb is in its native form and the heme group embedded in a hydrophobic pocket formed by the protein’s backbone by means of appropriate folding [23–32, 34–39]. The absorbance intensity of Soret band of Hb increases gradually with increasing concentration of Ag NPs (*C*_*Ag*_ = 100–600 µM) with a small red shifted (≈ 9.2 nm). The red shifting and change of absorption intensity of Soret band of Hb is found with increasing concentration of Ag NPs by means of the out plane distortions of the iron atom in the heme plane by disturbing the iron from heme-porphyrin moieties (Fig. 3) [40–47]. The change in intensity of tryptophan band, ε band, Soret band and Q-band of Hb with Ag NPs implies that Ag NPs interact with both heme and tryptophan residues.

**Fig. 4:**
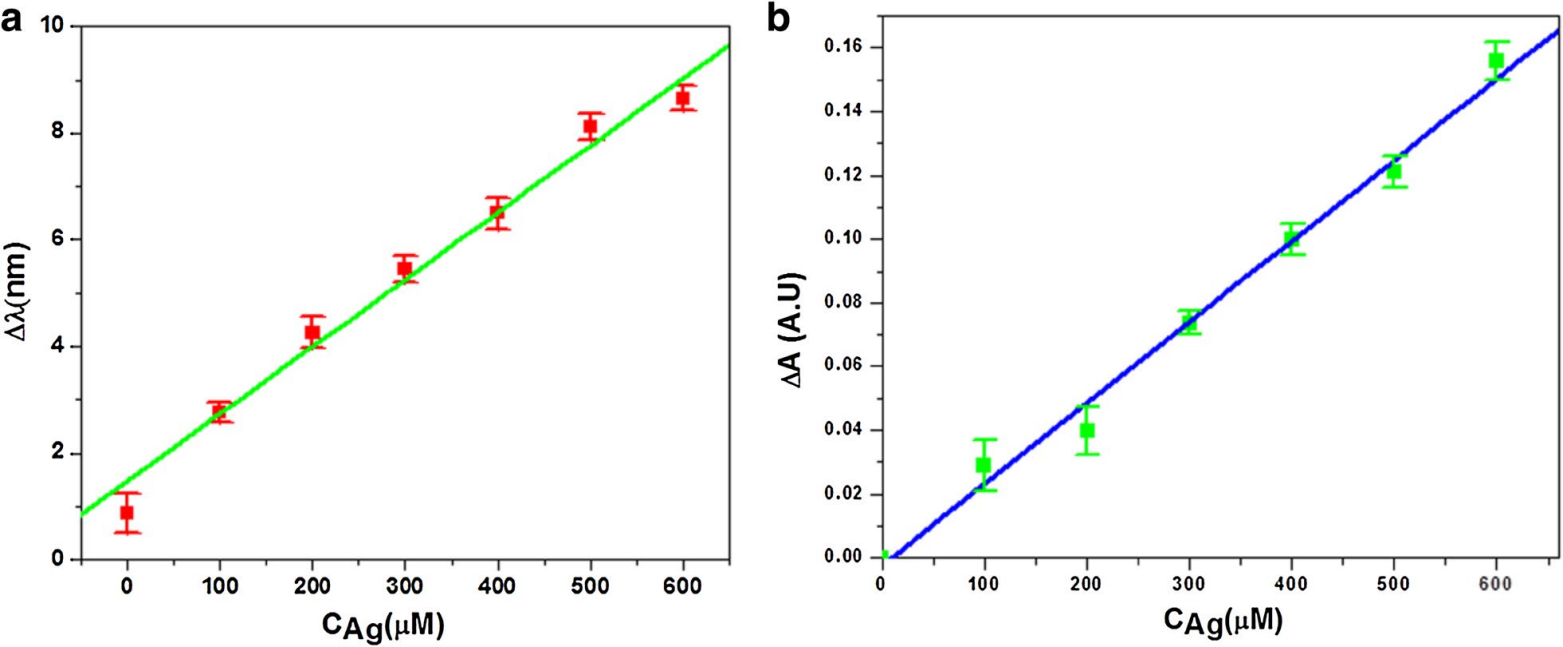
**a** Variation of change 1in the absorption peak wavelength (Δλ) of soret band with increasing concentration of silver nanoparticles (C_Ag_), **b** variation of change in the absorption peak intensity (ΔA) of soret band with increasing concentration of silver nanoparticles (C_Ag_)

Our results show that the absorbance at 280 nm of tryptophan (Trp) in Hb increases with increment of *C*_*Ag*_. The plots of *C*_*Ag*_ vs change in wavelength (∆*λ*) (Fig. 4a) and *C*_*Ag*_ vs change in absorbance (∆*A*) (Fig. 4b) illustrate that the interaction increases with increasing *C*_*Ag*_. The gradual increment of intensity of absorbance of Trp of Hb with *C*_*Ag*_ may be due to the formation of the ground state complex [41, 48]. The gradually red shifting of tryptophan’s absorption with increment of C_Ag_ confirms that Ag NPs perturbs Hb through interaction with Trp and heme-porphyrine moieties of Hb.

### Kinetic study of the adsorption of Hb to the AgNPs

**Fig. 5:**
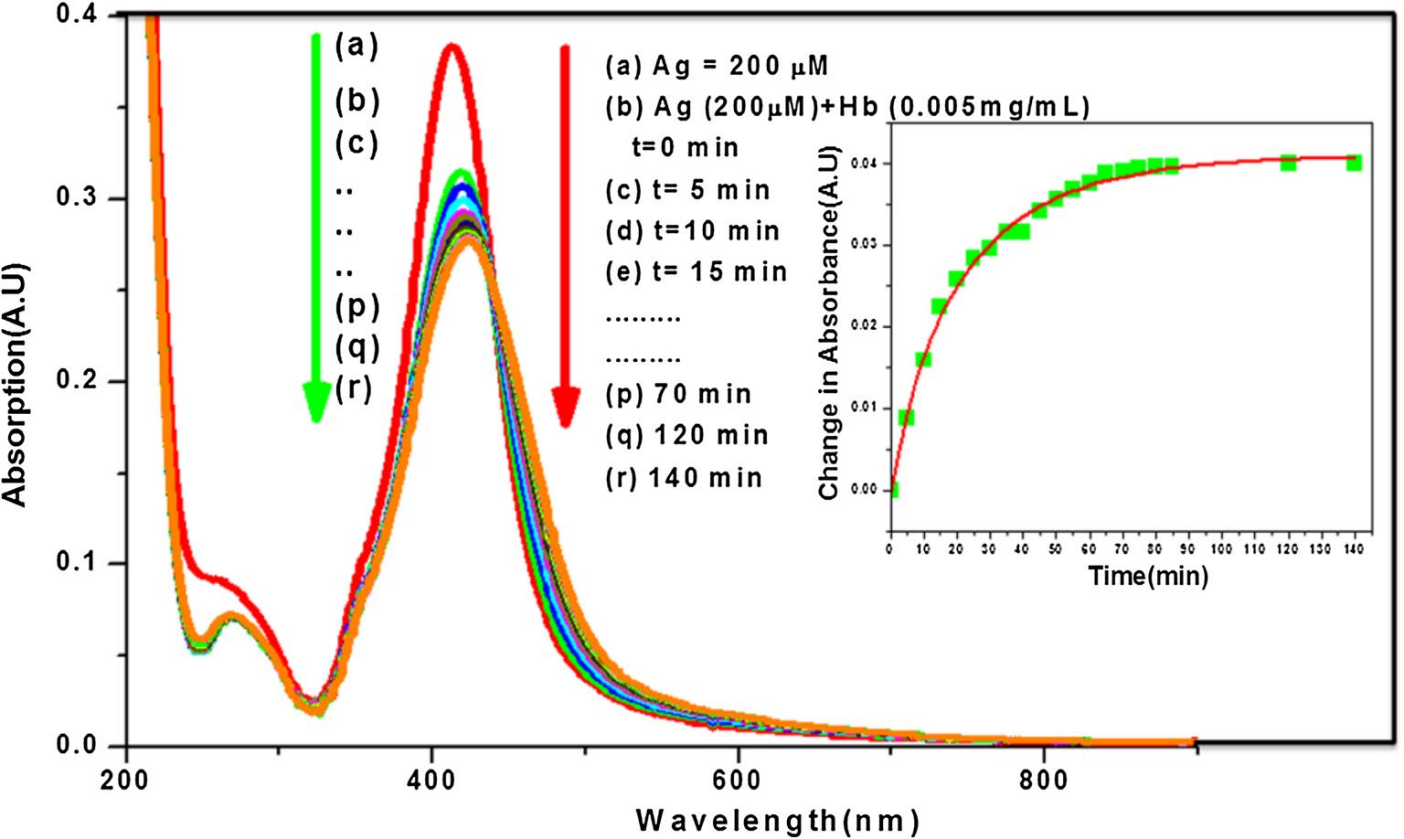
Time variation of SPR Absorption peak; Hb-Ag NPs complex with with C_Hb_ = 0.005 mg/mL and C_Ag_ = 200 µM. Inset shows the plot of change of SPR absorption intensity of Ag NPs with time. Red line shows the fitting curve fitted by Eq. 1

To analyze the rate of adsorption of Hb on the surface of Ag NP, we took benefite of the sensitivity of the longitudinal surface plasmon resonance (LSPR or SPR) band (at 410 nm) of the AgNP to the surrounding dielectric environment, and measured the change of Hb binding as a function of the change in the Plasmon absorption over a time scale of 0–140 min (Fig. 5). This kinetic analysis indicates that Hb binds to and saturates the surface Ag NPS after only several minutes. The Plasmon quenching is mainly due to plasmon resonance energy transfer (PRET) from nanoplasmic Ag NPs to adsorbed Hb molecules [49].

The kinetics of SPR due to Hb injection is studied by fitting the change of Plasmon peak intensity of Ag NPs with time by the following exponential association equation [50] (inset of Fig. 5).

1 $$I_{t} = I_{o} + A_{1} \left[ {1 - \exp \left( { - \frac{t}{{t_{1} }}} \right)} \right] + A_{2} [1 - { \exp }( - \frac{t}{{t_{2} }})]$$

where, I_o_ and I_t_ are the Plasmon peak (absorption) intensities at time zero and t, respectively due to binding with Hb. The constants A_1_ and A_2_ are the relative contributions and t_1_ and t_2_ are corresponding time constants of two mechanisms (surface binding and reorganization of Hb), respectively involving in Ag NPs–Hb interaction.

We have found, *t*_*1*_ = 9.51 min for the binding of Hb with surface of Ag NPs for the formation of corona and *t*_*2*_ = 118.48 min revels to reorganization of Hb due to binding with Ag NPs. The higher value of A_2_ (0.035) than A_1_ (0.006) implies that the Hb corona formation is more prominent than unfolding, which well agreement with other results (CD measurement, Zeta potential, DLS). The exponential association confirms that the formation of ‘Ag NPs–Hb’ corona starts immediately after incorporation of Ag NPs into Hb. The hemoglobin (Hb) needs a relatively long time to shield the original surface of Ag NPs and reorganization of Hb occurs [51, 52].

### Kinetics of Ag–Hb corona by autocorrelation function and dynamic light scattering (DLS) study

**Fig. 6:**
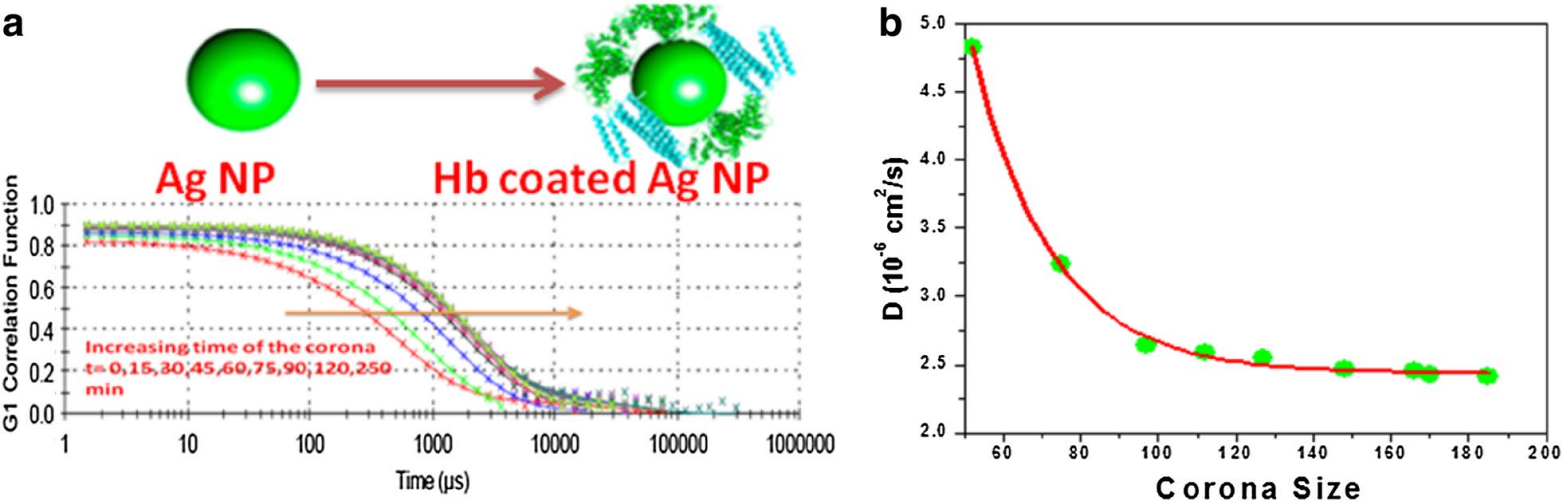
**a** Variation of Correlation Function of the Ag NPs–Hb composite under different time of the corona. **b** Variation of the diffusion coefficient with corona size under different time of the corona

The formation of Hb monolayer on the Ag NP surface leads to the increasing NP size due to Hb molecule adsorption. Hemoglobin adsorption leads to the formation of corona. The autocorrelation function is found to broaden with the increasing time of the formation of corona. The increasing Hb–AgNP corona size results in an increase of diffusion correlation time (τ_D_), indicated by a shift of the autocorrelation function curve toward longer times (Fig. 6). This implies time dependent corona decrease in the decay rate of the autocorrelation function and hence the decrease in the average diffusion coefficient [53]. The diffusion coefficient of the Hb–Ag NP in these systems has been calculated using the Stokes–Einstein relation (Eq. 2) [54].

2 $$D = \frac{{K_{B } T}}{{3\pi \eta d_{n} }}$$

where k_B_ is Boltzmann’s constant, d_n_ is the measured effective hydrodynamic diameter, T is absolute temperature and η is the viscosity of the solvent.

**Fig. 7:**
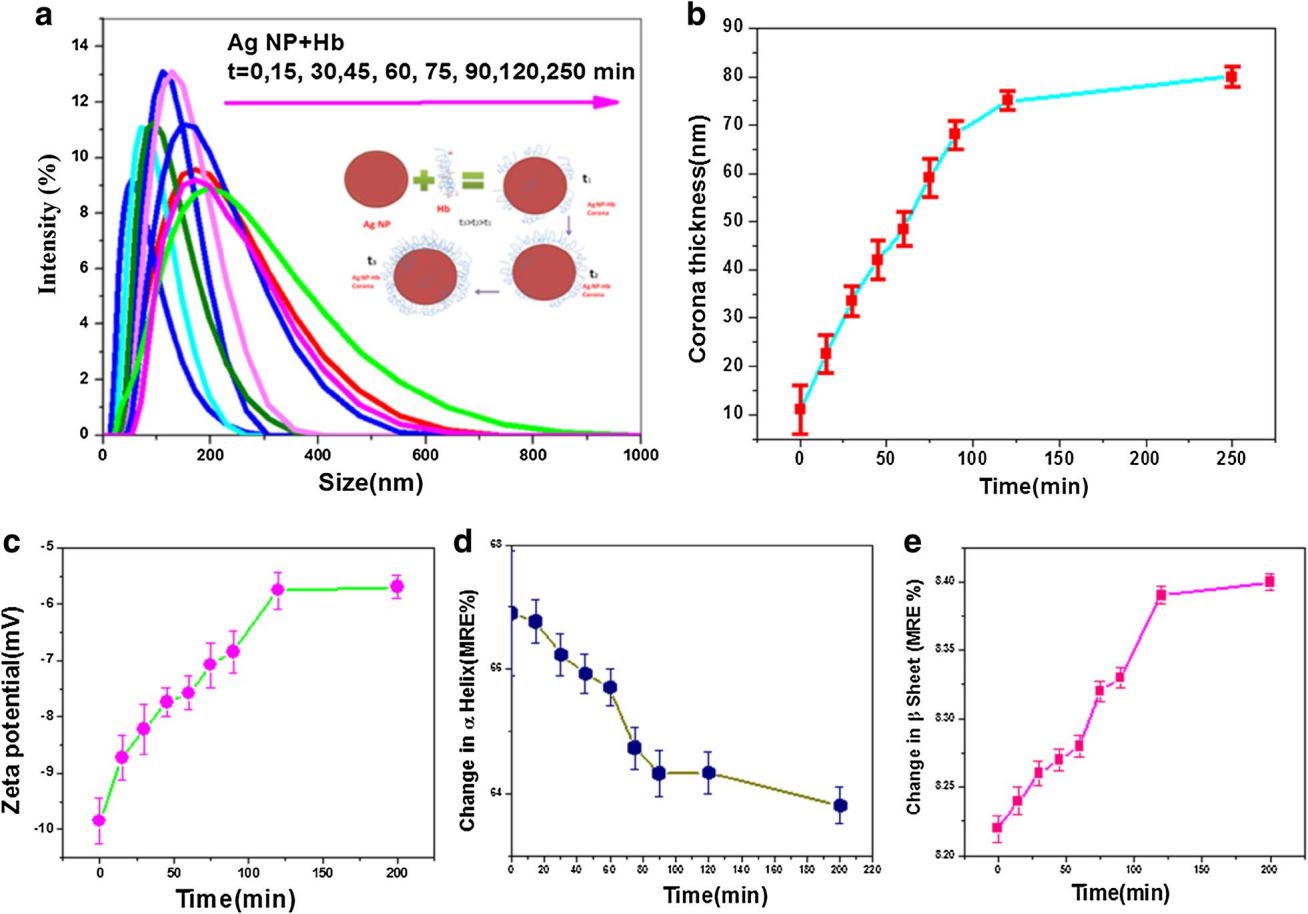
**a** Dynamic light scattering spectra of Ag NP-Hb corona under different time (0 to 250 min) of the corona, inset shows the schematic diagram of the time dependent corona, Time variation of Ag–Hb corona results: **b** Variation of Hb corona thickness with time. **c** Variation of zeta potential of the corona with time. **d** Change in α-helix of the Hb with time of the corona formation. **e** Change in β-sheet of the Hb with time of the corona formation due to interaction with Ag NPs

In our observation the decrease in diffusion coefficient with respect to Ag NP–Hb corona (Fig. 6b) could be because of an increase in Hb-induced attractive interaction between nanoparticles and/or due to nanoparticle aggregation [55]. The HRTEM visualization images supports this result. The dynamic light scattering (DLS) data of the Ag NP–Hb system are shown in Fig. 7a. In the size distributions of Ag NP–Hb corona at different time of the corona two important points are observed: (i) the average size of the Ag NPs–Hb corona increasing with increase in time of the corona and (ii) the broadening of the PSD (for higher time of the corona) at much higher sizes than that of the first size distribution. The increase in the average size of corona of the first size distribution can be explained by enhancement in attractive interaction between NPs and/or being due to emergence of the process of aggregation, whereas the second PSD corresponds to the large nanoparticle aggregates representing a two-phase system which support TEM results. This phase behavior of silver nanoparticles with oppositely charged Hb arises because of the strong electrostatic binding of the Hb with the Ag NPs [56, 57]. This results supports the zeta potential variation of the corona. Time variation of Hb corona thickness with time in Ag–Hb corona is shown in Fig. 7b.

#### Time dependent zeta potential of Ag–Hb corona

In order to reach deeper knowledge insight into the Hb-induced variations in net surface charge of Ag NPs, zeta potential measurements were carried out under similar experimental states as described above. The surface charge of NPs is directly related to the magnitude of electrical potential at the surface of NPs and the thickness of the corona layer [58, 59]. Upon incubating Ag NPs dispersion with Hb, the zeta potential value of Ag NPs showed a significant decrease from − 15.50 to − 5.50 mV over 120 min (Fig. 7c and Table 1). After then, there were no major variations in zeta potential is observed. The observed decrease in surface charge is attributed to the screening of negatively charged Ag NPs by surface-bound Hb molecules, which may establish electrostatic interactions between the surface ions and the positively charged amino acid residues of the Hb (histidine, lysine) [60, 61].

**Table 1:** Changes of the surfaces charges with the increase of the time of the corona

Time of the corona (min)	Average value of the surface zeta potential in mV
0	− 9.84
15	− 8.73
30	− 8.22
45	− 7.74
60	− 7.58
75	− 7.08
90	− 6.85
120	− 5.76
200	− 5.69

### Time dependent circular dichroism (CD) spectra

**Fig. 8:**
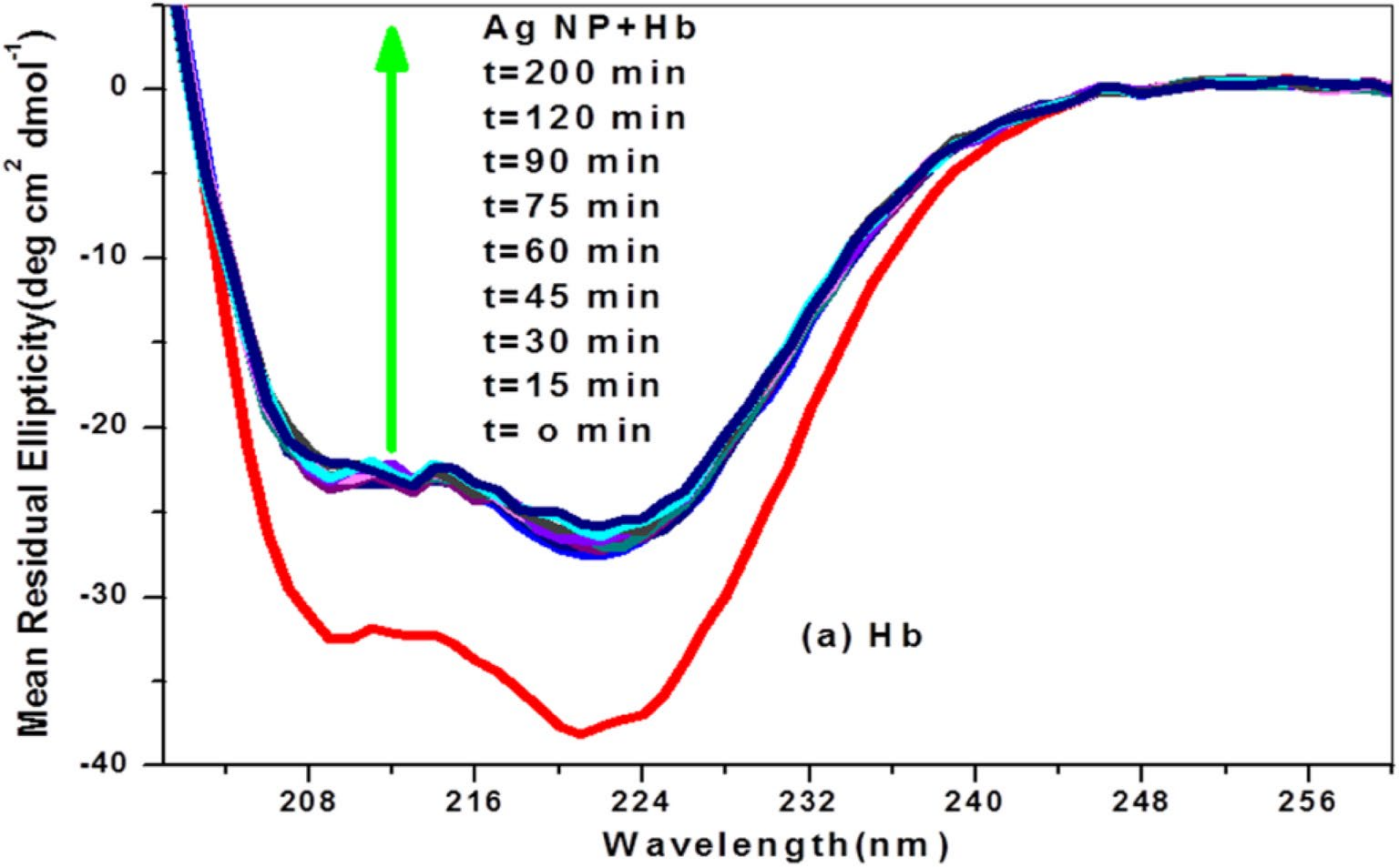
CD Spectra of pure Hb and Hb-Ag nanocomposite after different time of corona (from 0 to 200 min)

Figure 8 shows the typical CD spectra of Hb and Hb–Ag NPs corona at different time of the corona. The CD spectrum of native Hb (at pH 7.0) exhibited two negative bands in the far UV region (190–260 nm) at 208 and 222 nm [curve (a) in Fig. 8], which is characteristic of an α-helix structure of Hb. The band at 208 nm corresponds to π–π* transition of the α-helix, whereas the band at 222 nm corresponds to π–π* transition for both the α-helix and random coil. The addition of Ag NPs causes the decrease in the intensity of both the negative bands, with peak position remain same but slight distortion in shape. As is evident from the Fig. 8, the MRE values at 208 nm of Hb slight decreased with increasing time of the Ag NPs–Hb corona, indicating loss of the α-helical content of Hb after the conjugation. With the increasing of the time of the corona of AgNPs in the bioconjugate system, the intensities of the two bands increase further, and two bands appear to move together toward the region between 208 and 222 nm. As a result, the CD spectra evolve toward a shape more similar to that of typical β-sheet-rich structure, which is likely to be an indication of the conformational transition from α-helix to β-sheet structure in the bioconjugate system [59]. In this work, the secondary structural elements of Hb in native and in Hb-AgNPs bioconjugate system are calculated from CD data at pH 7.0 using K2D3 program. It is indicated that the native conformation of Hb contains 74.46% α-helix structures, 8.56% β-sheets [62]. After formation of the Hb–AgNPs bioconjugate system, the amount of α-helix decreases 63.8%, and β-sheet increase up to 8.42% along with increasing the time (200 min) of the AgNPs–Hb corona (Fig. 7c, d).

The change in α-helical content of Hb is not significant after a certain time of the corona, which implies that the protein could retain most of its helical structure after conjugation with AgNPs. Therefore, loss of secondary structure of the protein was expected for the basic form of Hb [63]. This change in the original structure of the protein may result in loss of its biological activity or the activation of immune response [64].

### Visualization of the hemoglobin corona by high-resolution transmission electron microscopy (HRTEM)

**Fig. 9:**
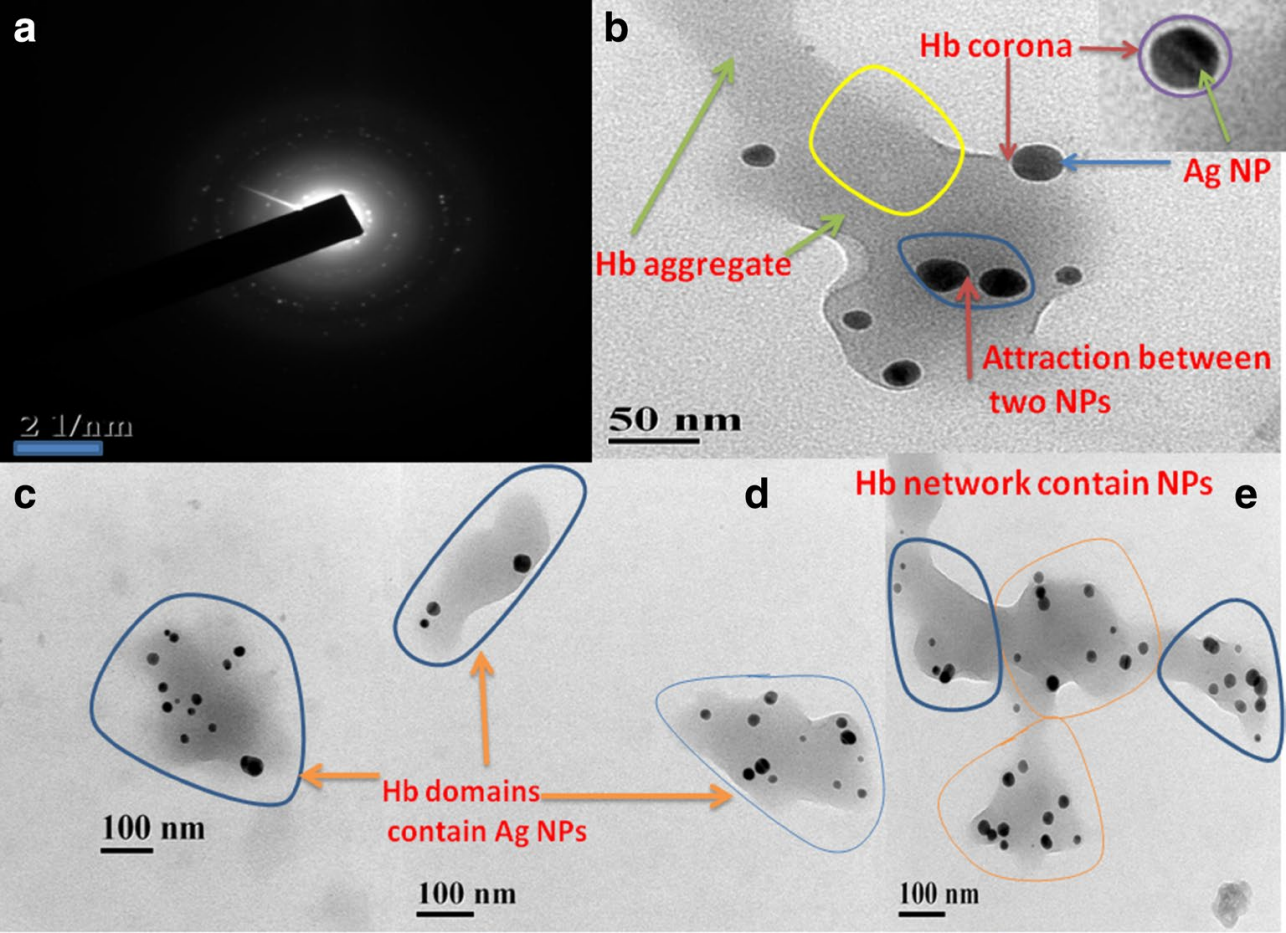
**a** SAED pattern of Hb-Ag corona (Amorphous in nature due to Hb and few bright spot due to Ag NPs inside the protein matrix), HR-TEM images Hb-Ag complex: **b** Aggregation of Hb after interaction and corona formation with Ag NPs, inset is the enlarge portion of Hb corona around single Ag NP. **c**, **d** Different Hb domains contain Ag NPs. **e** Different domains of Hb contains Ag NPs are connecting each other and form Hb network

Transmission electron microscopy (TEM) analysis revealed an even, thin shell of Hb corona formed over the AgNP (inset of Fig. 9b). More detailed structural images of the Hb–Ag nanoparticle assemblies (using TEM images) show both isolated Ag nanoparticles and clusters of Ag NPs placed within a Hb domain (Fig. 9b–e) with no apparent modification of the Ag nanoparticle morphology. In addition to these composite assemblies, large domains of Hb aggregates containing essentially no Ag NPs were also found. Such domains are linearly forming Hb network (Fig. 9e). This image clearly shows that Ag NPs (appearing as clear dark spots in the images) and Ag NPs aggregates are distributed throughout the Hb network (Fig. 9e).

Some of these Hb aggregates have well-defined profiles while others exhibit a random, amorphous morphology. The SAED pattern of the Hb conjugated Ag NPs shows amorphous in nature due to Hb and few clear dark spot due to Ag NPs inside the Hb matrix (Fig. 9a).

### Emission of Hb–Ag NPs corona under three different temperatures: fluorescence quenching study and energy transfer efficiency

**Fig. 10:**
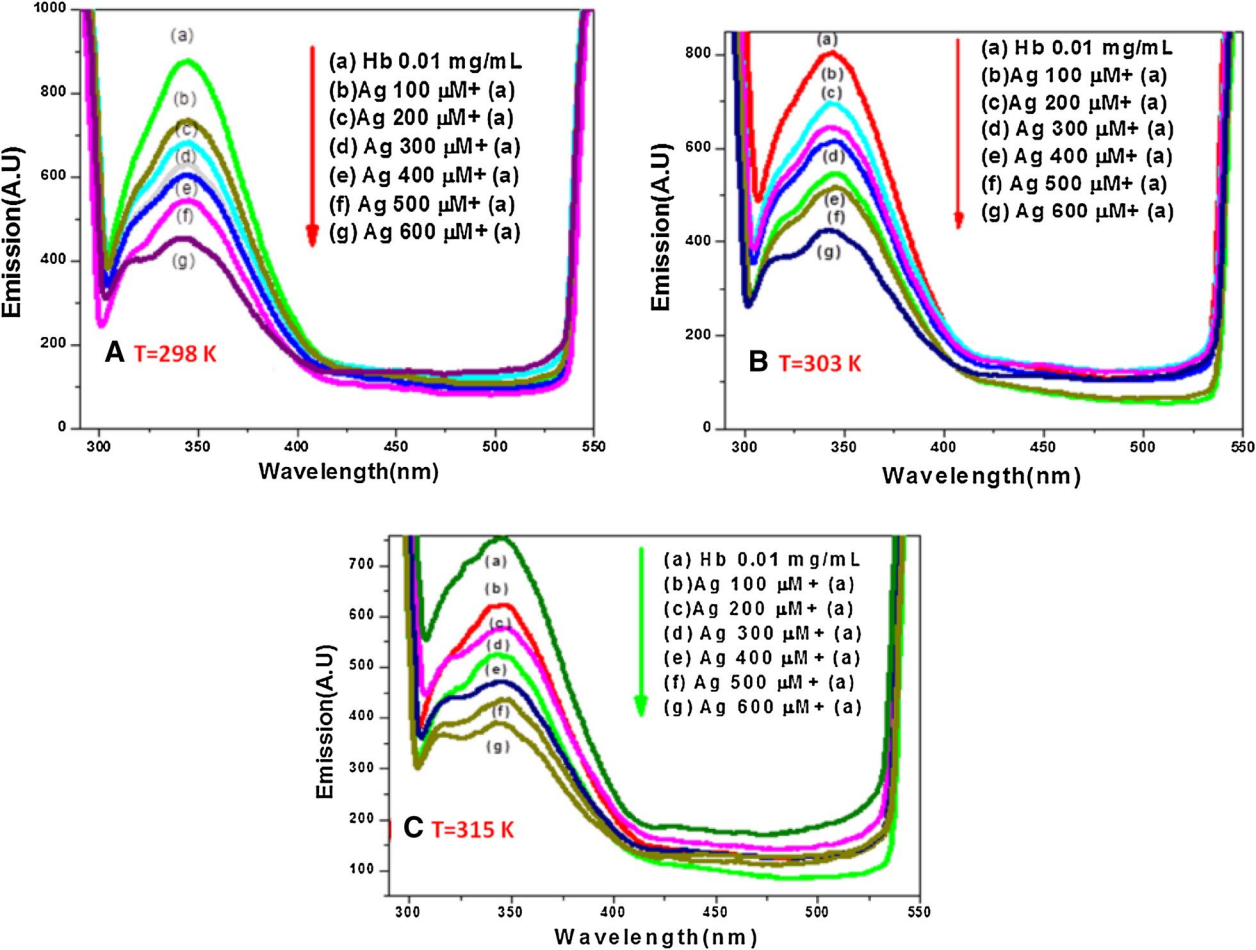
**A** (a) represents the fluorescence spectrum of pure Hb with C _Hb_ = 0.01 mg/mL and (**b**–**g**) represent the fluorescence spectra of Hb in Hb–Ag NPs complex with C_Hb_ = 0.01 mg/mL and C_AgNPs_ = 100, 200, 300, 400, 500, 600 µM at T = 298 K, **B** (a) represents the fluorescence spectrum of pure Hb with C_Hb_ = 0.01 mg/mL and (b)–(g) represent the fluorescence spectra of Hb in Hb–Ag NPs complex with C_Hb_ = 0.01 mg/mL and C _AgNPs_ = 100, 200, 300, 400, 500, 600 µM at T = 303 K. **C** (a) represents the fluorescence spectrum of pure Hb with C_Hb_ = 0.01 mg/mL and (b)–(g) represent the fluorescence spectra of Hb in Hb–Ag NPs complex with C_Hb_ = 0.01 mg/mL and C _AgNPs_ = 100, 200, 300, 400, 500, 600 µM at T = 315 K

The maximum fluorescence emission spectrum intensity (I_max_) of pure Hb arises around 340 nm. This emission (at 340 nm) corresponding to tryptophan residues of Hb. The addition of Ag NPs solution of different concentrations (*C*_*Ag*_ = 100–600 µM) to Hb solution (*C*_*Hb*_ = 0.01 mg/mL) at temperature 293, 303 and 315 K, results a decrease in the maximum fluorescence emission spectrum intensity (I_max_) around 340 nm with slide distortion in original shape. The reduction in the emission intensity and distortion of pure Hb emission (at 340 nm) suggesting the occurrence of fluorescence quenching process (Fig. 10A–C) [65]. This result confirms the denaturation of Hb structure. The binding of Hb with Ag NPs is examined by Stern–Volmer equation [66].

3 $$\frac{{I_{0} }}{I} = K_{SV} \left[ Q \right] + 1$$

**Fig. 11:**
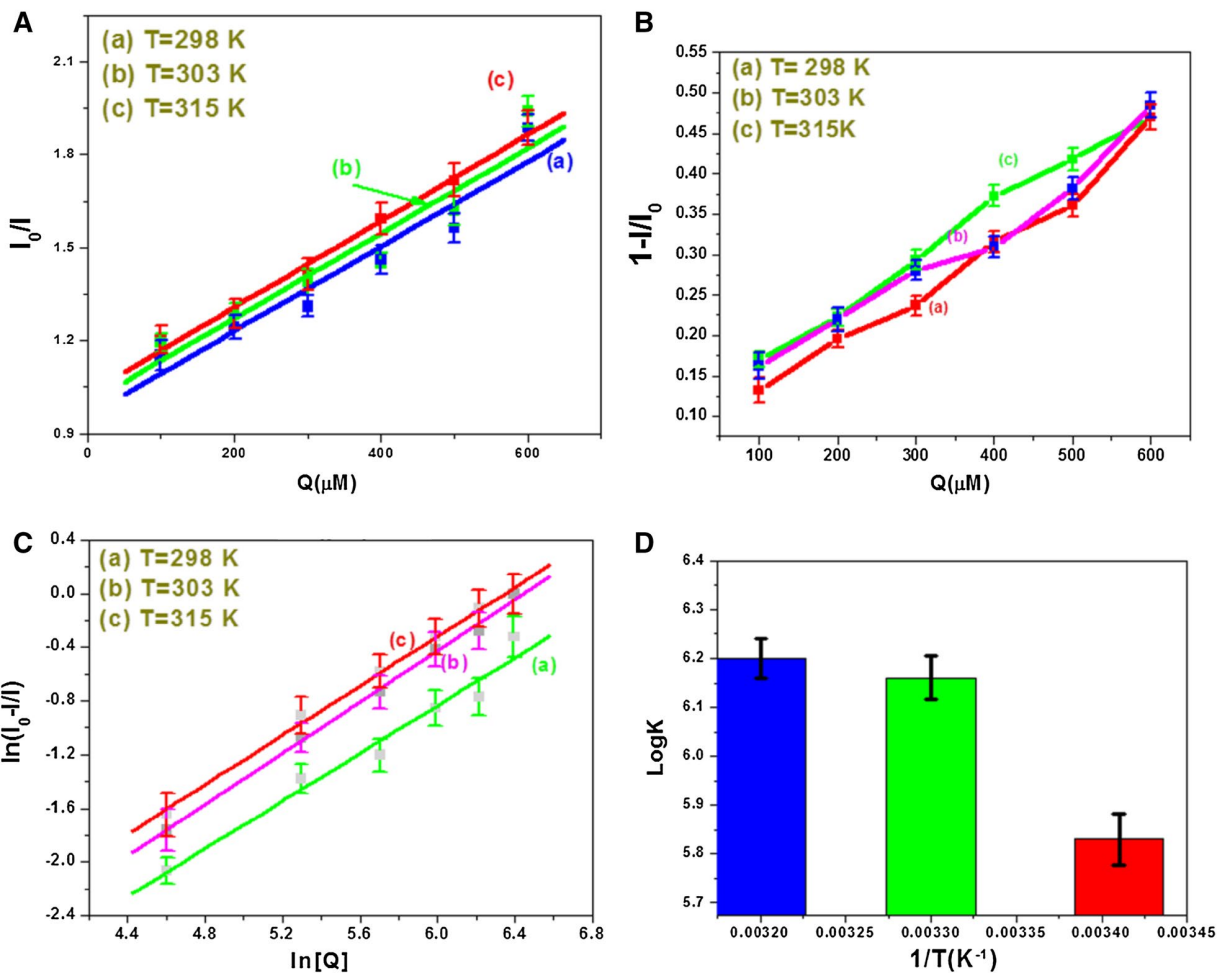
**A** represents the I_0_/I versus Q plot for Ag NPs-Hb complex at the temperatures (a) 298 K (b) 303 K (c) 315 K, **B** variation of energy transfer efficiency (1-I/I0) for (a) T = 298 K (b) T = 303 K and (c) T = 315 K due to interaction with BSA; **C** represents the log [I_0_− I/I] versus Log [Q] plot for Ag NPs-Hb complex at the temperatures (a) 298 K (b) 303 K (c) 315 K, **D** variation of log K versus 1/T plot (error bar diagram) for Ag NPs-Hb complex

In the present study the increment of *K*_*SV*_ (Stern–Volmer quenching constant) with increasing temperature from 293 to 315 K signifies that the quenching mechanism of Hb is dynamic in presence of AgNPs and the strength of interaction increases within this temperature range [59]. The I_0_/I versus [Q] plots at different temperatures are shown in Fig. 11A. The binding constant (K) and number of binding sites (n) between Hb and Ag NPs at different temperatures are calculated using the following equation:

4 $$\log \left[ {\frac{{I_{0} }}{I} - 1} \right] = logK + n { \log }[Q]$$

The variation of ${ \log }[\frac{{I_{0} }}{I} - 1]$ versus log [Q] is shown in Fig. 11C. The values of *K*_*SV*_, n, logK, at 293, 303, and 315 K are summarized in Table 2 for comparison. At the temperature 298 K, negative cooperative reaction takes place (n < 1). However, at the higher temperature [nearest to human body temperature (315 K)], the positive cooperative (n > 1) reaction is found.

**Table 2:** The thermodynamic parameters and binding parameters

Temp (K)	K_sv_ (M^−1^)	**n**	LnK (µM^−1^)	ΔG kJ/mol	ΔH (kJ/mol)	ΔS
298	1.37	0.91	5.835	− 14.428	− 99.19 kJ/mol	14.794 J/mol
303	1.39	1.02	6.161	− 15.494		
315	1.41	1.11	6.182	− 16.055		

Different thermodynamic parameters [change in enthalpy (∆*H*), change in entropy (∆*S*)] for Hb and Ag NPs interaction are studied using the van’t Hoff equation [66].

5 $$\ln K = - \;\frac{\Delta H}{RT} + \frac{\Delta S}{R}$$

Variation of log K versus 1/T plot is shown in Fig. 11D. The free energy change is analyzed by following relationship.

6 $$\Delta {\text{G}} = \Delta {\text{H}} - {\text{T}}\Delta {\text{S}} = -\; {\text{RTlnK}}$$

The resultant values of the thermodynamic parameters are summarized in Table 2 to account for the main forces contributing to the stability of Hb and binding mechanism. The negative value of Δ*G* indicates the spontaneity of the binding of Hb to Ag NPs. The negative value of Δ*H* represents that binding is an exothermic interaction process. The positive value of ∆S represents that electrostatic interaction between Trp of Hb and surface of Ag NP. This electrostatic force is the foremost force for binding of Ag NPs–Hb. After electrostatic interaction, the hydrophobic interaction takes place as evident from red shift of fluorescence peak of Hb in presence of Ag NPs.

The florescence resonance energy transfer efficiency between Ag NPs and Hb is measured using the following equation [53, 67–69].

7 $${\text{Q}}_{\text{eff}} = 1 - \frac{\text{I}}{{{\text{I}}_{0} }}$$

Here, I and I_0_ are the relative emission intensity of the Hb, in the absence and the presence of Ag NPs respectively. The energy transfer efficiency increases as the increase of concentration of the Ag NPs. The maximum calculated efficiency is around 48% corresponding to 600 µM of the Ag NPs (almost same for all three experimental temperatures). The variation of energy transfer efficiency with concentration of different temperature of the corona is shown in Fig. 11B.

## Conclusion

To the best of our Knowledge, this is the important report to study the time dependent interaction as well as the corona formation of Hb–Ag NPs bioconjugate. The red shift of SPR peak of Ag, in Ag NPs–Hb system, confirms the complex formation of Ag NPs with Hb. The results from the time dependent SPR absorption confirm the corona formation of Hb with the surface of Ag NPs through the formation of stable corona. The time constants for surface binding and reorganization are found 9.51 and 118.48 min, respectively. The hydrodynamic size of the corona along with zeta potential and SPR absorption of the Ag NP stops increasing at some point (after 120 min), support the idea of complete corona formation needs few hours. This Hb absorption process gives the idea about ultra high molecular sensitivity method. Thin shell of Hb corona formed over the AgNP and Ag nanoparticle aggregates are distributed throughout the Hb network leads to corona and deformation of Hb structure in time dependent manner as observed from HRTEM image and CD spectroscopy. The amount of α-helix decreases 63.8%, and β-sheet increase 8.42% along with increasing the time (200 min) of the corona resultant structural deformation of Hb is also found in presence of Ag NPs. The change of intensity of the fluorescence emission peak of the Hb in the Ag NPs–Hb conjugated system confirms the occurrence of fluorescence quenching and energy transfer. The quenching process occurs via interaction of the Trp residues accessible to the metallic surface of the Ag NPs. We have found that the Trp moieties are the most favorable binding sites of Hb with Ag NPs. The positive cooperative binding at lower temperature and negative cooperative binding at body temperature of Trp with Ag NPs induces the Hb molecules to organize at the surface boundaries of Ag NPs. Our Hb conjugate Ag NPs could cover the manner for scheming new optical based materials for the application in chemical sensing or biosensing. The Ag NPs–Hb interaction represents the fundamental of bioreactivity of metal nanoparticles. A significant increase in biomedical applications of Ag nanoparticles and their potential toxicity requires several studies by different way to determine Ag NPs-protein interactions. Our results are the awareness of the health effect of Ag nanoparticles.

The experimental details and characterization methods is included in the supporting section (Additional file 1).

## Additional file

**Additional file 1.** Experimental details and characterization methods.
