# Supplementary material for: Silver nanoparticle-human hemoglobin interface: time evolution of the corona formation and interaction phenomenon
Source: Nano Converg. 2017 Oct 30;4:28. doi: 10.1186/s40580-017-0122-1 (PMC5661023; doi:10.1186/s40580-017-0122-1)
Supplement: Supplementary file 1 — Additional file 1. Experimental details and characterization methods. [file 40580_2017_122_MOESM1_ESM.docx]

**Silver Nanoparticle- Human Hemoglobin Interface: Time Evolution of the Corona Formation and Interaction Phenomenon**

A. K. Bhunia^1,2,*^, T. Kamilya^3^ , S. Saha^1^

^1^Department of Physics & Technophysics, Vidyasagar University, Paschim Medinipur, -721102, India

^2^Department of Physics, Government General Degree College at Gopiballabhpur-II, Beliaberah, Paschim Medinipur-721517, India

^3^Department of Physics, Narajole Raj College, Paschim Medinipur-721211, India

**S1. Experimental details and Characterization methods**

The so prepared Ag NPs were dispersed in Millipore water and the concentration of the Ag NPs was varied from 100 µM to 600 µM. AgNPs-Hb mixed solution was prepared by mixing 0.01mg/mL Hb with 100 µM AgNPs with Ag NPs ranging from 100 µM to 600 µM with proper ratio. Also Hb-Ag NPs mixed solution were prepared by mixing 0.005mg/mL Hb with 200 µM Ag NPs.

The Hb solution with predetermined concentration of Hb (*C_Hb_=*0.001 to 0.08 mg/mL) was prepared by using triple distilled water, deionized with a Milli-Q water purification system from Millipore, U.S.A (pH and resistivity were 6.8 and 18.2 MΩ cm, respectively).

The optical absorption spectra of the samples were recorded in a Shimadzu-Pharmaspec-1700 UV-VIS spectrophotometer. The fluorescence spectra were obtained by using Perkin Elmer LS 55 spectrophotometer. For microstructural study, a small drop of water dispersed samples was placed on a thin carbon film supported on copper grid and kept for some time for drying. The high resolution transmission electron micrograph (HRTEM) of the prepared Ag NPs and Ag-Hb conjugate was acquired using JEOL-JEM-200 operating at 200kV. The SAED pattern of the said NPs was also carried out insitu with HRTEM. Dynamic Light Scattering (DLS) measurements were made with a Malvern Zeta Sizer Nano ZS instrument operating with a wavelength of 532 nm and a fixed scattering angle of 173°. The circular dichroism (CD) spectra of the samples were recorded by JASCO J-815 CD Spectrometer.
